# Supplementary material for: Collaborative intelligence in AI: Evaluating the performance of a council of AIs on the USMLE
Source: PLOS Digit Health. 2025 Oct 9;4(10):e0000787. doi: 10.1371/journal.pdig.0000787 (PMC12510544; doi:10.1371/journal.pdig.0000787)
Supplement: S2 File — (ZIP) [file pdig.0000787.s002.zip › S2 Data Raw data files containing unprocessed question inputs and LLM outputs including transcripts of deliberations/STEP3_Question-1-1_Type-MC-NJ.docx]

**Facilitator** **[Round 1]:** A 30-year-old woman comes to the office because of a 3-day history of joint pain in her hands and a rash over her chest and arms that is slowly resolving. The joint pain in her hands has persisted and is exacerbated by writing or typing. She rates the pain as a 3 on a 10-point scale. The patient is a preschool teacher and reports that one of her students had a facial rash and fever 1 week prior to the development of her symptoms. The patient's medical history is unremarkable and her only medication is an oral contraceptive. She is in a monogamous relationship with her husband. She does not smoke cigarettes, drink alcoholic beverages, or use illicit drugs. BMI is 22 kg/m² . Vital signs are temperature 38.1°C (100.5°F), pulse 94/min, respirations 18/min, and blood pressure 107/58 mm Hg. Physical examination discloses a blanching, erythematous, papular rash on the anterior chest and proximal upper extremities. The first and second metacarpophalangeal joints of both hands are tender and swollen. The wrist joints also are tender to palpation but there is minimal swelling. Which of the following microorganisms is most likely to have caused this patient's symptoms? (A) Adenovirus (B) Borrelia burgdorferi (C) Coxsackievirus (D) Ehrlichia chaffeensis (E) Parvovirus B19

**AI_A:** (1) RESTATING THE QUESTION: A 30-year-old woman, a preschool teacher, has been experiencing a three-day bout of pain in her hand joints and a slowly subsiding rash over her chest and arms. She mentioned that a student exhibited a facial rash and fever about a week before her symptoms manifested. Clinical examination revealed a blanching, erythematous, papular rash on her upper body and the first and second metacarpophalangeal joints of both hands are tender and swollen. The question aims to deduce which microorganism from the list provided - Adenovirus, Borrelia burgdorferi, Coxsackievirus, Ehrlichia chaffeensis, Parvovirus B19 – may have provoked the woman's symptoms. (2) PLANNING: The root assumptions are that the patient's presentation is due to an infectious disease and that the infectious agent is one of the options given. To generate alternate assumptions, one might question whether her symptoms could be the result of an allergy, or an autoimmune disorder. Given her contact with preschool children and the timing of her symptoms following the student's illness, it is reasonable to prioritize the assumption of an infectious disease. (3) CLINICAL REASONING: In examining each microorganism, Adenovirus typically causes respiratory illness, not joint or rash symptoms. Borrelia burgdorferi causes Lyme disease, which does involve rash and joint symptoms, but the rash is typically a target or "bull's-eye" lesion, not described here. Coxsackievirus can cause hand-foot-mouth disease, notable for painful sores in the mouth and a rash on the hands and feet. However, this patient's symptoms are hand joint pain, not sores, and a rash on her chest and arms, not her hands and feet. Ehrlichia chaffeensis brings on human monocytotropic ehrlichiosis, characterized by fever, headache, muscle aches, and sometimes a rash, but not typically joint pain and swelling. Parvovirus B19 tends to cause "fifth disease," which features a facial rash, fever, and joint symptoms, typically in adults. This patient's rash and joint symptoms match those of fifth disease, and her recent exposure to a child with a facial rash and fever supports a diagnosis of a parvovirus B19 infection. (4) CONCLUDING: The clinical picture, with a rash over the chest and proximal extremities, joint pain and swelling, and exposure to a child with similar symptoms, best aligns with infection by Parvovirus B19, known to cause fifth disease. (5) RESPONSE: Consequently, I infer that it is Parvovirus B19 (E) from the list of microorganisms provided that is most likely to have caused the patient's presenting symptoms.

**AI_D:** RESTATING THE QUESTION: The issue at hand involves determining which microorganism is likely to be the causative agent for a 30-year-old woman's presenting symptoms. The patient reported to a clinic with joint pain that has lasted for 3 days, worsened by activities such as writing, and a resolving rash covering her chest and arms. Recently, one of her preschool students had a fever and facial rash. No significant findings were found in her medical history apart from her use of oral contraception. Her lifestyle does not involve smoking, alcohol consumption, or illicit drug use. Professional examination revealed a blanching, reddish, papular rash on her chest and upper extremities' proximal aspects. Additionally, there was tenderness and swelling in the metacarpophalangeal joints of her hands, along with tender wrists. PLANNING: Initial assumptions might include that her symptoms are due to an illness that is easily transmitted, considering her profession and connection to a student exhibiting similar symptoms. Nonetheless, this conjecture requires further validation. To generate alternative assumptions, one needs to deliberate whether or not her symptoms could have been a result of ailment from her significant other, environmental factors, or even her using oral birth control pills. Upon evaluating these assumptions, giving clinical priority to the conjecture regarding an infectious agent appears to be most logical due to her profession as a preschool teacher, her close contact with children, and the recent symptomatic case of her student. CLINICAL REASONING: Given the connection with the symptomatic student and the nature of the symptoms - joint pain, rash resolving slowly, and low-grade fever, the involvement of a viral pathogen is highly plausible. In this context, Parvovirus B19 stands out due to its potential to cause erythema infectiosum, or "Fifth disease," commonly seen in children, which can present with a facial rash (slapped cheek) and fever. This disease is known to affect adults, primarily presenting as arthralgias and arthritis, often in the metacarpophalangeal joints, exactly as described in this scenario. CONCLUDING: The sudden emergence of the symptoms, their specific traits, and the link to her student strongly indicate a viral infection. Furthermore, the joint pain, resolving rash, and her occupational exposure to a likely reservoir of infection (children in a preschool), the signs strongly suggest an infection with Parvovirus B19. RESPONSE: Thus, considering the above-mentioned clinical reasoning, the microorganism most likely responsible for this female patient's symptoms is (E) Parvovirus B19.

**AI_C:** 1) RESTATING THE QUESTION: Allow me to summarize, our patient, a healthy 30-year-old woman who is a preschool teacher, is presenting with joint pain in her hands that worsens with writing or typing, and a resolving rash on her chest and arms. She reports exposure to a student who had a facial rash and fever a week before the onset of her symptoms. There is evidence of infection as she has a low-grade fever, and her rash is blanching (lessening or disappearing under pressure), erythematous (red or reddish), and papular (small, raised, solid pimple-like), concentrated on her anterior chest and upper extremities. The joints in her hands and wrists are tender and swollen. We are tasked to identify the microorganism most likely causing her symptoms. 2) PLANNING: To decide the microorganism causing her symptoms, it's important to consider her exposure history, symptom profile, and pattern of disease presentation. Often, direct contact or encounter is a common route of transmission for most of these options. Additionally, rashes associated with fever or systemic symptoms might suggest a viral etiology. 3) CLINICAL REASONING: Restating my thoughts, if we look into each proposed microorganism: (A) Adenovirus commonly presents with conjunctivitis, pharyngitis, and gastroenteritis - symptoms that our patient doesn't have; (B) Borrelia burgdorferi, the causative agent for Lyme disease, often presents with erythema migrans, but this patient does not exhibit its characteristic 'bulls-eye' rash or any other related symptoms like headache, fatigue, and potential neurological or heart involvement; (C) Coxsackievirus typically presents with a febrile illness associated with sore throat and chest pain, which doesn't quite align with our patient's presentation; (D) Ehrlichia chaffeensis, the causative agent for human monocytic ehrlichiosis, usually associated with ticks, presents with nonspecific symptoms of fever, headache, and myalgia, without the joint pain and rash indicated here. 4) CONCLUDING: Based on the symptom (e.g., joint pain), examination (e.g., erythematous papular blanching rash), and patient history (e.g., exposure to a sick pupil), (E) Parvovirus B19, which can cause erythema infectiosum (a common viral rash illness in children also known as the fifth disease), appears most consistent with the patient's presentation. Commonly seen symptoms include a 'slapped cheek' rash, joint pain, and a lacy rash on chest and arms – all of which have appeared in this case. 5) RESPONSE: The most likely microorganism causing symptoms in this patient appears to be (E) Parvovirus B19.

**AI_E:** (1) RESTATING THE QUESTION: The case is about a 30-year-old woman who, in the past three days, has experienced joint pain in her hands and a slowly resolving rash on her chest and arms. She works as a preschool teacher and had come into contact with a student experiencing a facial rash and fever a week earlier. She has no significant medical history, and her only medication is an oral contraceptive. She is in a healthy sexual relationship with her husband. She doesn't consume alcohol, cigarettes, or illicit drugs. She has a normal body mass index and has presented with a slightly elevated temperature. On physical examination, she has a blanching, erythematous, papular rash and tender, swollen metacarpophalangeal and wrist joints. Now, we are attempting to identify the likely causative microorganism of her symptoms. (2) PLANNING: Each of the potential causative agents listed has certain associated clinical features: (A) Adenovirus: Causes respiratory illnesses (B) Borrelia burgdorferi: Causes Lyme disease which presents with erythema migrans, not a papular rash. (C) Coxsackievirus: Causes hand-foot-mouth disease, herpangina - would typically present with mouth sores and a rash on hands and feet too, not primarily the anterior chest and proximal upper extremities. (D) Ehrlichia chaffeensis: Causes ehrlichiosis, usually includes fever, headache, fatigue, and muscle aches. (E) Parvovirus B19: Causes erythema infectiosum, can also result in ill-defined systemic symptoms, arthritis or arthralgias and, in some cases, a rash. Taking the patient's symptoms into account, Parvovirus B19 appears to be the most probable cause, but questioning assumptions such as the timing of the symptoms and the lack of any significant medical history are essential while reasoning clinically. (3) CLINICAL REASONING: The fact that the patient's student had a facial rash and fever one week prior to her development of symptoms could suggest a communicable disease. Given the patient's symptoms, Parvovirus B19 is a promising alternate assumption as it is common among children and can cause symptoms such as joint pain, fever, and rash in adults. This virus, in particular, is known for causing erythema infectiosum also known as "fifth disease," which typically presents with a facial rash and often spreads to the limbs which matches the clinical history of the student and the patient. (4) CONCLUDING: Evaluating the different microorganisms, the natural progression of the symptoms (especially the spread of the rash from the student to the teacher), and the professionally exposed environment, Parvovirus B19 seems to be the most likely cause of the patient's symptoms. There appears to be a strong correlation between the symptoms of erythema infectiosum (fifth disease) in the student and the current condition of the patient. Primary infection with Parvovirus B19 often results in arthritis or arthralgias especially in adults, which correlates with the patient's painful hand joints. (5) RESPONSE: Given the pattern of the rash, the joint pain mainly in the hands, and the patient's contact history with her student who had a similar rash and fever, the most likely causative microorganism for this patient's symptoms is Parvovirus B19 (E).

**AI_B:** RESTATING THE QUESTION: The clinical presentation indicates a young woman who works as a preschool teacher experiencing joint pain and a resolving rash, with a recent exposure to a child with a facial rash and fever. She has no significant medical history and leads a healthy lifestyle. On examination, she has erythema and tenderness in her hand and wrist joints and a blanching rash spreading from her chest to her upper extremities. The challenge here is to identify the microorganism responsible for her signs and symptoms, among the options given, which include Adenovirus, Borrelia burgdorferi, Coxsackievirus, Ehrlichia chaffeensis, and Parvovirus B19. PLANNING: The potential causes for her symptoms among the mentioned organisms are in the framework of either viral or bacterial infections. Adenovirus could cause general symptoms like fever, but not a particular joint pain or resolution rash. Borrelia burgdorferi causes Lyme disease with a characteristic rash and joint symptoms but usually manifests with an 'erythema migrans' rash and history in tick-infested areas. Coxsackievirus generally causes hand, foot, and mouth disease which presents with characteristic mouth ulcers and hand/foot rashes, not a chest extending rash. Ehrlichia chaffeensis can cause ehrlichiosis, a condition that can present with a rash but typically also includes other symptoms like confusion and muscle aches, which are absent here. Parvovirus B19, however, can cause fifth disease, which presents with a "slapped-cheek" rash (similar to a facial rash in her student) and can cause a 'lace-like' rash and joint symptoms, specifically in adults. CLINICAL REASONING: While initial consideration might cause Borrelia burgdorferi (due to joint inflammation and rash) to be a likely cause, the rash in Lyme disease is characteristically an expanding erythema migrans lesion with central clearing, which does not match her blanching rash that appears to resolve. Also, significant Lyme arthritis, which primarily affects knees, would be unusual with only a three-day illness and no mention of tick exposure. The Ehrlichia chaffeensis usually presents with fever, headache, and confusion, which are absent in this case presentation. Coxsackievirus causes Hand, foot, and mouth disease with ulcers in mouth and rashes on distal extremities, which do not match her presentation. Adenovirus most commonly causes respiratory illness and would be unlikely to account for this presentation. Among the options, Parvovirus B19 is known to cause fifth disease or erythema infectiosum, a common condition among school-age children, is recognizable by initial fever, headache, and "slapped cheek" rash, common in children, followed by migration of the rash on the chest and upper extremities, which might be resolving and look "lace-like" in nature. Adults can also experience mild joint pain and swelling, most notably in the hands, as is seen in this patient. CONCLUDING: Based on the unique association between the presentation of a rash, joint pain in adults, and recent exposure to a child with a possible "slapped cheek" rash, Parvovirus B19 should be clinically prioritized as the cause of this patient's symptoms. RESPONSE: Thus, the microorganism most likely to have caused this patient's symptoms is (E) Parvovirus B19.

**Facilitator** The consensus statement synthesizes the responses of AI_A, AI_B, AI_C, AI_D, and AI_E as follows: The case involves a 30-year-old woman, a preschool teacher, who has been experiencing joint pain in her hands and a slowly resolving rash over her chest and arms for three days. She reported that a student had a facial rash and fever about a week before her symptoms appeared. Her medical history is unremarkable, and she leads a healthy lifestyle. The clinical examination revealed a blanching, erythematous, papular rash on her upper body and tender, swollen metacarpophalangeal joints in her hands. The task is to identify the microorganism most likely responsible for her symptoms from the options provided: Adenovirus, Borrelia burgdorferi, Coxsackievirus, Ehrlichia chaffeensis, and Parvovirus B19. The initial assumption is that her symptoms are due to an infectious disease, given her contact with preschool children and the timing of her symptoms following the student's illness. Each microorganism was examined for its typical symptoms and disease presentation. Adenovirus usually causes respiratory illnesses, not joint or rash symptoms. Borrelia burgdorferi causes Lyme disease, which involves rash and joint symptoms, but the rash is typically a target or "bull's-eye" lesion, not described here. Coxsackievirus can cause hand-foot-mouth disease, notable for painful sores in the mouth and a rash on the hands and feet, but this patient's symptoms are hand joint pain and a rash on her chest and arms. Ehrlichia chaffeensis causes human monocytotropic ehrlichiosis, characterized by fever, headache, muscle aches, and sometimes a rash, but not typically joint pain and swelling. Parvovirus B19, however, is known to cause "fifth disease," which features a facial rash, fever, and joint symptoms, typically in adults. This patient's rash and joint symptoms match those of fifth disease, and her recent exposure to a child with a facial rash and fever supports a diagnosis of a parvovirus B19 infection. The clinical picture, with a rash over the chest and proximal extremities, joint pain and swelling, and exposure to a child with similar symptoms, best aligns with infection by Parvovirus B19. Therefore, the consensus is that the microorganism most likely to have caused the patient's presenting symptoms is Parvovirus B19.
